# Molecular Identification of *Dibothriocephalus nihonkaiense* Infection Using Nanopore Sequencing: A Case Report and Literature Review

**DOI:** 10.3390/diagnostics14242871

**Published:** 2024-12-20

**Authors:** Hin-Fung Tsang, Stanley W.M. Leung, Tin-Nok Hung, Icy Law, Ka-Wai Lam, Loiston Chan, Sze-Chuen Cesar Wong

**Affiliations:** 1 Department of Clinical Laboratory and Pathology, Hong Kong Adventist Hospital, Hong Kong SAR, China; 2 Department of Applied Biology & Chemical Technology, The Hong Kong Polytechnic University, Hong Kong SAR, China

**Keywords:** *Diphyllobothrium nihonkaiense*, *Dibothriocephalus nihonkaiense*, fish tapeworm, diphyllobothriasis, next-generation sequencing, nanopore, epigastric pain

## Abstract

**Background:***Dibothriocephalus nihonkaiense* (previously known as *Diphyllobothrium nihonkaiense*) infection is not common in Hong Kong. *D. nihonkaiense* is a fish-borne cestode parasite that infects humans after consuming raw or insufficiently cooked fish containing plerocercoids. **Case presentation:** We reported a case of *D. nihonkaiense* infection in a 40-year-old woman who presented with a complaint of epigastric pain and diarrhea. A curvilinear opacity was seen at the upper quadrant of the abdomen via abdominal X-ray. An incomplete 80 cm long strobila of *D. nihonkaiense* without a scolex and neck was found in her feces. A grayish-brown oval egg with an inconspicuous operculum and small knob at the abopercular end was also found. Species-level identification was performed using Nanopore sequencing. Complete blood count and serum vitamin B12 level were tested to check for megaloblastic anemia and vitamin B12 deficiency, respectively. Laboratory investigations demonstrated an elevated percentage of monocytes in peripheral blood. A single oral dose of praziquantel (25 mg/kg) was prescribed to the patient. There was no evidence of relapse after the treatment. **Conclusions:** We reported a case of *D. nihonkaiense* infection using Oxford Nanopore NGS as a tool for accurate parasite identification.

## 1. Introduction

*Dibothriocephalus nihonkaiense* (previously known as *Diphyllobothrium nihonkaiense*), also known as broad fish tapeworm, is one of the largest intestinal tapeworms (the maximum length up to 25 m) infecting humans and other mammals [1,2]. Humans are infected after consuming raw or insufficiently cooked fish containing the plerocercoids of *D. nihonkaiense*. A tapeworm consists of three major parts: scolex, neck and strobila. *Dibothriocephalus* spp. are characterized by the presence of a scolex with a paired slit-like attachment groove (bothrium) on the dorsal and ventral surfaces. The neck is present posterior to the scolex. Strobila is a linear structure composed of a large number of proglottids, in which each segment contains the genital organs of both sexes [2]. There are at least 14 species of *Dibothriocephalus* spp. that can cause human diphyllobothriasis. Amongst them, *D. latus* and *D. nihonkaiense* are the main pathogens of humans, causing diphyllobothriasis [2]. Although diphyllobothriasis is known to occur worldwide, it is not common in Hong Kong. The second intermediate host of *D. latus* is freshwater fish such as pike, perch, burbot and char. *D. latus* infections are usually associated with cold waters. It is common in temperate regions where cold, clear lakes are abundant, such as Europe and North America [2]. As for *D. nihonkaiense*, its intermediate host is Pacific salmon. The consumption of raw Pacific salmon may be a risk factor for *D. nihonkaiense* infection. Although the geographical distribution of the intermediate hosts of *D. latus* and other *Dibothriocephalus* spp. such as *D. nihonkaiense* are different, because of marketing globalization, fish may be transported from their original fishing grounds to other selling locations. As a result, the imported parasite species may vary according to trade between countries, and hence the geographical distribution of *Dibothriocephalus* spp. is no longer area-restricted. Due to the high morphological similarity, it is challenging to distinguish *D. latus* from *D. nihonkaiense* accurately solely using morphology-based diagnostics, and molecular identification of the parasite becomes crucial for accurate species identification.

In Asia, *D. nihonkaiense* infections have been reported in the Republic of Korea [3] and Japan [4]. Some cases were also reported in Taiwan [1], Siberia [5] and Malaysia [6]. Most patients with *D. nihonkaiense* infections are asymptomatic but some may have gastrointestinal symptoms such as abdominal discomfort, diarrhea and weight loss. Many patients become aware only when the proglottids of the *D. nihonkaiense* are excreted. Common clinical manifestations of *D. latus* infection include megaloblastic anemia and vitamin B12 deficiency in the case of prolonged infection [7]. However, those clinical manifestations are not commonly seen in patients infected by other *Dibothriocephalus* spp. In this case report, we reported a case of *D. nihonkaiense* infection in a woman with recurrent epigastric pain. Species-level identification was performed using Nanopore sequencing because of its fast real-time sequencing capabilities.

## 2. Case Presentation, Materials and Methods

### 2.1. Case Presentation

**Figure 1:**
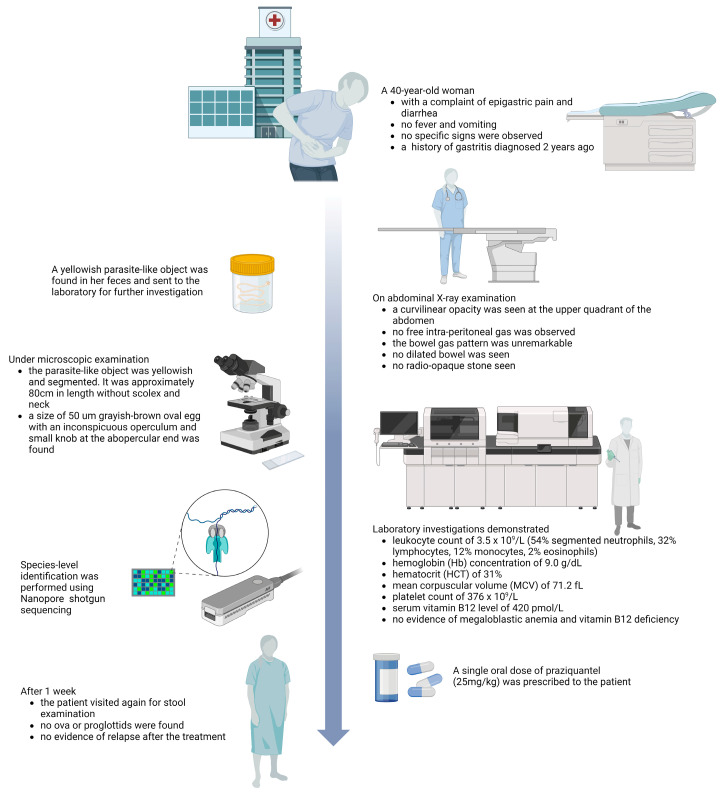
A summary of the case presentation. This figure was created with BioRender.com.

A 40-year-old woman was presented to the outpatient department with a complaint of epigastric pain and diarrhea. The patient had no fever and vomiting. On physical examination, no specific signs such as abdominal tenderness were observed. This patient has a history of gastritis diagnosed two years ago. On abdominal X-ray examination, a curvilinear opacity was seen at the upper quadrant of the abdomen. No free intra-peritoneal gas was observed. The bowel gas pattern was unremarkable and no dilated bowel was seen. There was no radio-opaque stone seen. Figure 1 summarizes the case presentation.

### 2.2. Laboratory Investigations

**Figure 2:**
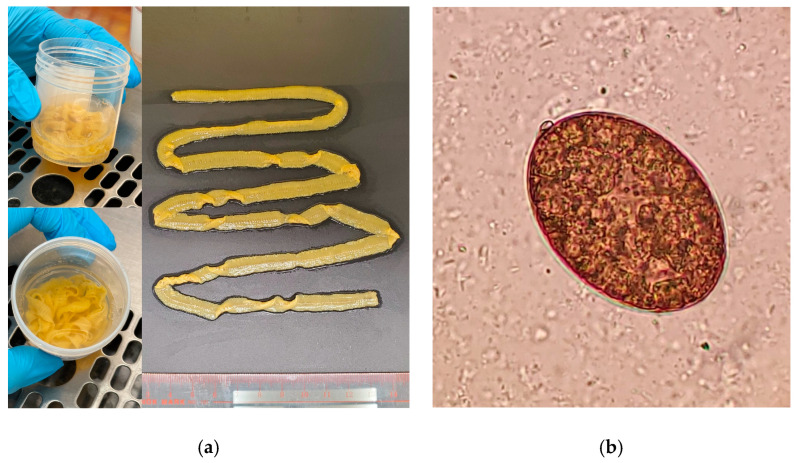
(**a**) The incomplete strobila of *Dibothriocephalus nihonkaiense* without scolex and neck (80 cm in length). The proglottids are more broad than long; (**b**) grayish-brown oval *Dibothriocephalus nihonkaiense* egg with a small abopercular knob (400×).

Complete blood count and serum vitamin B12 level were tested to check for megaloblastic anemia and vitamin B12 deficiency, respectively. Laboratory investigations demonstrated hemoglobin (Hb) concentration of 9.0 g/dL (11.5–15.4 g/dL); leukocyte count of 3.5 × 10^9^/L (54% segmented neutrophils, 32% lymphocytes, 12% monocytes, 2% eosinophils) (3.7–9.3 × 10^9^/L); hematocrit (HCT) of 31% (34–46%); mean corpuscular volume (MCV) of 71.2 fL (80–96 fL); platelet count of 376 × 10^9^/L (160–420 × 10^9^/L); and serum vitamin B12 level of 420 pmol/L (145–569 pmol/L). There was no evidence of megaloblastic anemia and vitamin B12 deficiency. A yellowish parasite-like object was found in her feces and sent to the laboratory for further investigation. On examination, the parasite-like object was yellowish and segmented. It was approximately 80 cm in length without scolex and neck (Figure 2a). A 50 um grayish-brown oval egg with an inconspicuous operculum and small knob at the abopercular end from the parasite was found under microscopic examination (Figure 2b).

### 2.3. Nanopore Sequencing

Shotgun sequencing was performed to accurately identify the parasite at species level. In brief, DNA was extracted from the proglottids tissue using the Qiagen TissueLyser III (cat. 9003240) (Qiagen, Hilden, Germany) and the Qiagen QIAamp DNA Blood Mini Kit (cat. 51104) (Qiagen, Hilden, Germany). The tissue was disrupted at 30 Hz for 15 s after being submerged in Buffer AL. Proteinase K was added, followed by incubation at 56 °C for 2 h. The DNA extraction was performed using the QIAamp kit according to the manufacturer’s instructions. Since the DNA was eluted in Buffer AE, 3x bead clean-up was performed using Roche KAPA Pure beads (cat. 07983271001) (Roche, Basel, Switzerland) to eliminate any residual EDTA that could potentially interfere with downstream processes.

Native genomic DNA was prepared using the Oxford Nanopore Technologies Ligation Sequencing Kit (SQK-LSK114) (ONT, Oxford, UK) following the manufacturer’s protocols, with the exception of the incubation times, which were doubled. The prepared DNA library was sequenced on an Oxford Nanopore Technologies MinION R10.4.1 flow cell using the MinION Mk1C sequencer.

The sequencing data were basecalled using the Dorado super accuracy basecaller model V4.3.0, optimized for a read length of 400 bases per second (dna_r10.4.1_e8.2_400bps_sup@v4.3.0). The resulting fastq data were subsequently uploaded to Chan Zuckerberg ID (CZID), a cloud-based metagenomics platform. Data analysis with CZID was conducted using the Nanopore mNGS Pipeline v0.7 with default parameters that include human genome filtering.

## 3. Results

**Figure 3:**
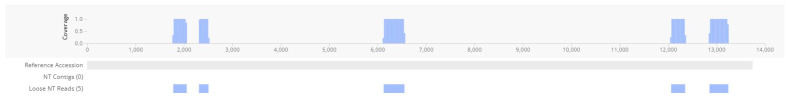
The coverage of the complete genome of *Dibothriocephalus nihonkaiense* mitochondrion (NCBI Reference Sequence: NC_009463.1).

An analysis using the CZID pipeline gave an average percent identity of the alignments to NCBI NT/NR (%id) of 94.3% and an average expected value of alignments to NCBI NT/NR (E-value) of 10^−181^, using the complete genome of *D. nihonkaiense* mitochondrion (NCBI Reference Sequence: NC_009463.1) as the reference. Five reads were found to be aligned to the taxon in the NCBI NT/NR database (Figure 3). The detailed parameters obtained by CZID are shown in Table 1 and the sequences obtained by nanopore sequencing are shown in Supplementary Document S1.

Taking into account the morphological characteristics of the strobila and egg, together with the DNA sequences of the mitochondrial genome obtained by Nanopore sequencing, the parasite-like object was identified as *D. nihonkaiense*. A single oral dose of praziquantel (25 mg/kg) was prescribed to the patient. The patient visited again after 1 week for stool examination and no ova or proglottids were found. There was no evidence of relapse after the treatment.

## 4. Discussion

*Dibothriocephalus* spp. infection is the cause of diphyllobothriasis. The consumption of raw and wild-caught fish prepared as sushi and sashimi is the major risk factor for diphyllobothriasis. Most cases of diphyllobothriasis are asymptomatic but some patients may have gastrointestinal symptoms such as abdominal discomfort, diarrhea and weight loss. Diphyllobothriasis is not common in Hong Kong and mainland China. In mainland China, only 22 cases of human diphyllobothriasis were reported between 1927 and 2014 [8,9].

The misidentification of the parasite may introduce errors in understanding the geographical distribution of the parasite, clinical presentation, pathogenesis and the epidemiology of the parasitic diseases. This is because different species of the same genus or family may have a different intermediate host and reservoir, clinical presentation and geographical distribution [10]. However, parasites are often incorrectly reported as the most common or most well-recognized species that infect humans instead of the actual species infecting the patient due to inadequate expertise [10]. *Dibothriocephalus* spp. is one of the most commonly misidentified parasites [10]. The accuracy of species identification of *Dibothriocephalus* based on morphological examination varies because inadequate morphology experience of the technician may lead to misidentification and inaccurate diagnosis [7]. The life cycle of *Dibothriocephalus* spp. is complex. It involves a wide diversity of freshwater and marine fish as the intermediate hosts, which are commonly pike, perch, burbot and char for *D. latus* and Pacific salmon (e.g., *Oncorhynchus masou*, *O. keta*, *O. gorbuscha* and *O. nerka*) for *D. nihonkaiense*. Due to the high morphological similarity, it is challenging to distinguish *D. latus* from *D. nihonkaiense* accurately using solely morphology-based diagnostics. Moreover, due to marketing globalization, fish may be transported from their original fishing grounds to other selling locations. As a result, the imported parasite species may vary according to trade between countries, and hence the geographical distribution of *Dibothriocephalus* is no longer area-restricted [11]. The molecular identification of the parasite is crucial for accurate species identification. However, many commercial and laboratory-developed tests may not detect all medically important parasites [10]. Moreover, although molecular assays such as PCR designed for distinguishing individual *Dibothriocephalus* species have been developed, they are not widely available in routine laboratory testing. Thus, sequencing-based methods have emerged as promising diagnostic tools and are necessary to confirm the species to understand the distribution and origin of infection of *Dibothriocephalus* spp. during epidemiological investigations. Recently, next-generation sequencing (NGS) has emerged as a promising diagnostic tool for the detection of any potential pathogens in various species types [12]. In this case report, an unbiased shotgun NGS using the Oxford Nanopore platform was used to identify the parasite at the species level. Oxford Nanopore sequencing technology is the fourth generation of sequencing technology, which allows the rapid identification and detection of infectious diseases [13]. Compared to first-generation sequencing technology such as Sanger sequencing, Nanopore sequencing allows fast real-time sequencing for the rapid diagnosis of infectious diseases. The portability of the Nanopore system, inexpensive sequencing device, relatively simple library preparation procedures and the real-time onboard basecalling make Nanopore sequencing suitable for on-site applications [14]. To the best of our knowledge, this case report is the first report using Oxford Nanopore sequencing technology in the species identification of *Dibothriocephalus* spp. This case also highlights the use of Oxford Nanopore NGS as a tool for accurate onsite parasite identification when accurate morphological diagnosis is not applicable.

Clinically speaking, human diphyllobothriasis may not cause obvious symptoms or may be associated with vague gastrointestinal symptoms only [7]. Many patients become aware of the infection only when the proglottids of the *Dibothriocephalus* spp. are excreted. The severity of the disease is associated with the worm burden and the by-products produced by *Dibothriocephalus* spp. In some cases, *Dibothriocephalus* infection can be long-lasting, potentially lasting for up to 25 years. In some rare cases, massive infections may cause intestinal obstruction and the aberrant migration of proglottids can cause cholecystitis or cholangitis. Vitamin B12 deficiency has been reported as a complication of prolonged *D. latus* infection as a result of the parasite-mediated dissociation of the vitamin B12-intrinsic factor complex within the human gut lumen, making vitamin B12 unavailable to the host [2]. Mild anemia or eosinophilia may also occur. However, those clinical manifestations are not commonly reported in patients infected by *D. nihonkaiense*. In our case, the serum Vitamin B12 level of the patient was within normal intervals.

In some reported cases of *D. latus* infections without molecular identification of the parasites, vitamin B12 deficiency was not observed in those patients. Molecular methods should be used to identify *Dibothriocephalus* spp. at the species level accurately, especially in regions where the intermediate host of *D. latus* is not commonly found. Clinical presentation, geographical distribution and the epidemiology of the parasitic diseases must also be considered in order to perform accurate parasite identification and diagnosis. In terms of treatment, a single oral dose of praziquantel (25 to 50 mg/kg) is usually prescribed and is highly effective against *Dibothriocephalus* spp. infections. *D. nihonkaiense* is more sensitive to praziquantel than *D. latus* [2]. The side effects of praziquantel are usually mild and require no treatment. An alternative anti-helminthic drug for human diphyllobothriasis is Niclosamide (a single dose of 2 g for adults and 1 g for children older than 6 years). However, the availability of Niclosamide is limited in many countries [2]. The plerocercoids of *Dibothriocephalus* spp. can be killed by cooking the fish at a temperature of 55 °C for 5 min [5] or freezing below −20 °C for 7 days or −35 °C for 15 h [15]. However, the smoking method does not kill *Dibothriocephalus* spp. [16]. Therefore, *Dibothriocephalus* spp. infection can be prevented by eating well-cooked or deep-frozen fish.

## 5. Conclusions

In conclusion, we reported a case of *D. nihonkaiense* infection in a woman with recurrent epigastric pain in Hong Kong. This case also highlights the use of Oxford Nanopore NGS as a tool for accurate parasite identification when accurate morphological diagnosis is not applicable.

## Figures and Tables

**Table 1:** Detailed parameters obtained by Chan Zuckerberg ID (CZID). The coverage of the complete genome of *Dibothriocephalus nihonkaiense* mitochondrion (NCBI Reference Sequence: NC_009463.1) by Nanopore sequencing.

Aligned Loose Reads	1	2	3	4	5
**Reference alignment range**	1779–2058	2311–2508	6126–6555	12,064–12,354	12,857–13,246
**Alignment length**	283	199	437	298	397
**Percentage matched**	96.1%	97.0%	92.2%	92.3%	94.0%

## Data Availability

The data presented in this study are available on request from the corresponding author.

## Supplementary Materials

The following supporting information can be downloaded at https://www.mdpi.com/article/10.3390/diagnostics14242871/s1, Document S1: The sequences of *Dibothriocephalus nihonkaiense* mitochondrion obtained by Nanopore sequencing.
