# Supplementary material for: Molecular Identification of Dibothriocephalus nihonkaiense Infection Using Nanopore Sequencing: A Case Report and Literature Review
Source: Diagnostics (Basel). 2024 Dec 20;14(24):2871. doi: 10.3390/diagnostics14242871 (PMC11675249; doi:10.3390/diagnostics14242871)
Supplement: Supplementary file 1 [file diagnostics-14-02871-s001.zip › diagnostics-3310494-supplementary.pdf]

>family_nr:-300:family_nt:28843:genus_nr:-200:genus_nt:2267273:species_nr:-
100:species_nt:2567821:NR::NT:NC_009463.1:05b08aaf-6aee-42aa-8516-6aee034e3de0
ATCCCCACTTTGGAAAAGTGATATAATTAAATTATACTAATAACATTAAGGTTACTTCAAAAGAAAACTAGCAGTACAAA
GTGATATAATCTTGACAAGAGTATACTATACACAACTCCCTAAACAAGTTGAAAGAGTATACTATACACAACTCCCTAAA
CAAGTTGAAAGAGTATCTATACACAACTCTCTAAACAA
>family_nr:-300:family_nt:28843:genus_nr:-200:genus_nt:2267273:species_nr:-
100:species_nt:2567821:NR::NT:NC_009463.1:133a5151-104b-472a-b6f4-78d50ea39b78
ATATTTGTACCTGGTTCAGTTGGCGTCTTGCTCTAAATCTCTGTGAACACGTGCAAACATCACTAATGATCTACTGAAAA
TGATAATATGCTAGTGACAATGTATCAGTGTACTAACGCTACAAAAACTTCGTAGAAAAAAACAACACCTAAAACTAATA
ACCACCATCAATTTATAACACAGAATTGTCCTACAGCAACAGCGCCAAAGCACCCTAATCTTAAATTGATAAAGAAAAGT
AGAAAGATCATCACAAAAGAACAAATAACATAACTGATAGTTTCTGCACAACAAACAGAGGACATATATATATAGTGAAT
ATACACGGAACGAATTTTATAAAGAATTCATGAGGAGAATTAGAAAAACGATTAATAGCAAACTCACAAAACAAAAAAAA
ACATAACTGACTAGACCACTGCGAACAAATGGTGATAAAACATATGAAACGGTAACTTGAGCGATACGTTGGT
>family_nr:-300:family_nt:28843:genus_nr:-200:genus_nt:2267273:species_nr:-
100:species_nt:2567821:NR::NT:NC_009463.1:33d8cc65-dbad-4adb-a9c1-4aeb50638d1e
ATGTTAGCACGACTACGTATTGCTTACAGGGGCAATTGTAGATGAAACTTATGTCCGCAACAGTTGAATAACAATTGATT
ACCATACTATTCCAAATAGTTTCTATTATTATTGCCTACTTCCTTTATGCCAGAGTGTTTAGGACAGATGATGAAGAAGT
TCACTATTTGGTTCAGATAACCTGGTTGAATATTTTTATGCTTCTTTTAATAGATTGCGTATCATTCTTAGTCAATTCTA
TTATCGTTGGGATAAGGAATGTATATCCTTATTAGCCAACAGTGCGAAATACCTAACAGTGCGTAAAGCAATACGTTA
>family_nr:-300:family_nt:28843:genus_nr:-200:genus_nt:2267273:species_nr:-
100:species_nt:2567821:NR::NT:NC_009463.1:976082ea-f82d-425d-919d-a7c25d7beb92
GATATACACCAACATAAACAGGATTAGATTAATAGGCGGTACGTCCTTTATAACACACCTTCCCCCTGTGATGGAACGTC
ACTCCCAAACTAATTTAGTTATAAAACTAAGTTATATTAAATGATTAATTAATGGGGTATCTAATCCCTTTCATAACCTA
ATCACTTATTAACGTATAAAATATTAGTGATTATAATAGAAAAAAATTTCACCTAATTTTATTTATAATTATTTTAAAAC
GTAACCACAAAGAGGCAAAACAGAATAACCGCGGATGCTGGCACTGTGCCAGCAATACAT
>family_nr:-300:family_nt:28843:genus_nr:-200:genus_nt:2267273:species_nr:-
100:species_nt:2567821:NR::NT:NC_009463.1:b97255c5-3a44-40fb-bfb0-3601a6553373
AGTTATTATGCTACAATACAACAAAACAGGTTTACTTTATAACTTCTATTACAATAGGCATATACCCGTGACCAACACCG
CATAACTCGCTACAATAACCCACAAAGACCCCATAGCGATCAGGCACAAAAAACAAAGATTTTATACGTCCTGGGATAGC
ATCCATCTTAATTTGAAGTAAATGATGCACAGAAGAAATGAATGAATAACATCCTCTGAGGTAACTAAAAACAATTAAAC
AACCCCATAATGTAATGGTAACGGCTTATCCACATGAAACCCATCCTTACATAAAAAAGAATCAAAAGAGCCCCCTGAAA
AATCATAAGACCAATATCATTGCGATTCCCACTACCTTAATTGTCTCCTGGAATGATCTAACCCGCGGTAAAGCAATACA
T
